# The Antidiabetic Drug Metformin Regulates Voltage-Gated Sodium Channel Na_V_1.7 via the Ubiquitin-Ligase NEDD4-2

**DOI:** 10.1523/ENEURO.0409-21.2022

**Published:** 2022-03-03

**Authors:** Alexandru-Florian Deftu, Paul Chu Sin Chung, Cédric J. Laedermann, Ludovic Gillet, Marie Pertin, Guylène Kirschmann, Isabelle Decosterd

**Affiliations:** 1 Pain Center, Service of Anesthesiology, Lausanne University Hospital (CHUV) and University of Lausanne, 1011 Lausanne, Switzerland; 2 Department of Fundamental Neurosciences, Faculty of Biology and Medicine, University of Lausanne, 1005 Lausanne, Switzerland

**Keywords:** dorsal root ganglion, metformin, Na_V_1.7, NEDD4-2, pain

## Abstract

The antidiabetic drug metformin has been shown to reduce pain hypersensitivity in preclinical models of chronic pain and in neuropathic pain in humans. Multiple intracellular pathways have been described as metformin targets. Among them, metformin is an activator of the adenosine 5′-monophosphate protein kinase that can in turn modulate the activity of the E3 ubiquitin ligase NEDD4-2 and thus post-translational expression of voltage-gated sodium channels (Na_V_s). In this study, we found that the bulk of the effect of metformin on Na1.7 is dependent on NEDD4-2. In HEK cells, the expression of Na_V_1.7 at the membrane fraction, obtained by a biotinylation approach, is only reduced by metformin when cotransfected with NEDD4-2. Similarly, in voltage-clamp recordings, metformin significantly reduced Na_V_1.7 current density when cotransfected with NEDD4-2. In mouse dorsal root ganglion (DRG) neurons, without changing the biophysical properties of Na_V_1.7, metformin significantly decreased Na_V_1.7 current densities, but not in *Nedd4L* knock-out mice (*SNS-Nedd4L*^−/−^). In addition, metformin induced a significant reduction in NEDD4-2 phosphorylation at the serine-328 residue in DRG neurons, an inhibitory phosphorylation site of NEDD4-2. In current-clamp recordings, metformin reduced the number of action potentials elicited by DRG neurons from *Nedd4L^fl/fl^*, with a partial decrease also present in *SNS-Nedd4L*^−/−^ mice, suggesting that metformin can also change neuronal excitability in an NEDD4-2-independent manner. We suggest that NEDD4-2 is a critical player for the effect of metformin on the excitability of nociceptive neurons; this action may contribute to the relief of neuropathic pain.

## Visual Abstract

**Figure:**
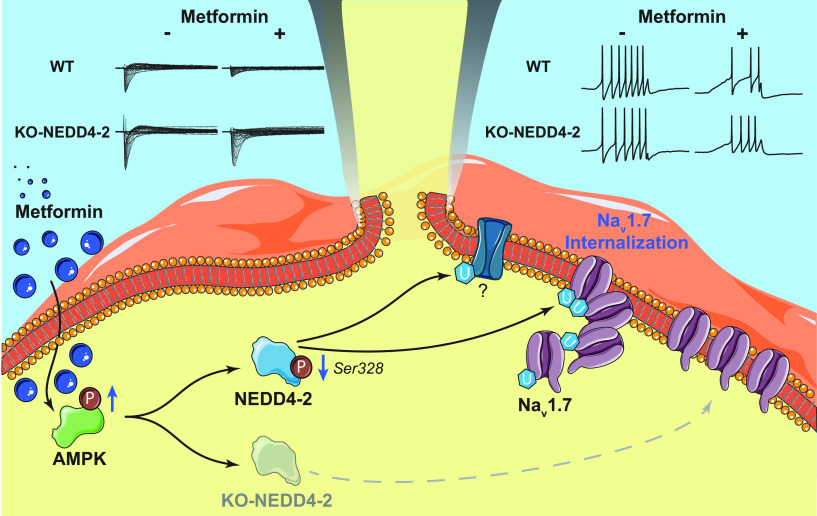

## Significance Statement

Metformin is a multitarget, antidiabetic drug that has shown therapeutic potential to reduce neuropathic pain. The intracellular mechanisms leading to a reduction in hyperexcitability and pain hypersensitivity remain unclear. We found that metformin acts via the activity of the E3-ubiquitin ligase NEDD4-2 to reduce cell surface expression and currents of voltage-gated sodium channels (Na_V_s), especially the Na_V_1.7 isoform. In current-clamp experiments, metformin reduced the DRG neuron firing frequency, with a smaller effect in knock-out mice (*SNS-Nedd4L*^−/−^). On the other hand, NEDD4-2 is indispensable for the metformin effect on the rheobase and the resting membrane potential of DRG neurons. These results suggest that NEDD4-2 activity is a crucial actor, although not exclusive, to reduce the excitability of nociceptive neurons.

## Introduction

Metformin is a antidiabetic drug with pleiotropic effects. Although it has been used for many decades as a first-line treatment to lower hyperglycemia and glycosylated hemoglobin in type 2 diabetes (Sterne, 1957; Hundal et al., 2000), its primary mechanism of action and effect on pain were found later. Zhou et al. (2001) showed that metformin induces the activation of adenosine 5′-monophosphate protein kinase (AMPK) in hepatocytes, a phenomenon that in turn regulates transcription factors and downstream signaling pathways, which was confirmed in humans (Musi et al., 2002). Patients with diabetes treated with metformin show less radiculopathy pain (Taylor et al., 2013) and musculoskeletal pain (Carvalho-e-Silva et al., 2021). In patients with fibromyalgia, metformin treatment significantly improved clinical symptoms such as pain, fatigue, depression, disturbed sleep, and tender points (Bullón et al., 2016).

One of the first studies indicating a potential use of metformin in experimental neuropathic pain showed reduction of tactile allodynia-like behavior in mice after spared nerve injury (SNI) and spinal nerve ligation (SNL; Melemedjian et al., 2011). In parallel, there was inhibition of the protein kinase complex mechanistic target of rapamycin (mTOR) complex 1 and activation of the ERK (extracellular signal-regulated protein kinase) pathways, ultimately leading to the dysregulation of translational control (Melemedjian et al., 2013). In rats, metformin reversed the chronic constriction injury-induced changes in phosphorylated signal transducer and activator of transcription 3 in spinal dorsal horn (SDH) and activation of microglia and astrocytes in the SDH, as well as decreased pain-like hypersensitivity (Ge et al., 2018). Metformin also prevented cisplatin-induced or spinal cord injury mechanical and thermal hypersensitivity (Mao-Ying et al., 2014), and attenuated the levels of tumor necrosis α and interleukin 1β in the spinal cord (Afshari et al., 2018).

Metformin acts via a multitude of intracellular signaling pathways in which the master kinase seems to be AMPK. This kinase is activated by an increase in the cytosolic AMP/ATP ratio, playing a crucial role in cellular energy homeostasis. A downstream ubiquitin protein ligase activated by AMPK is neuronal precursor cell expressed developmentally downregulated-4 type 2 (NEDD4-2), a ligase that acts on the epithelial sodium channel(ENaC) by downregulating its currents via ENaC–NEDD4-2-induced internalization (Bhalla et al., 2006). This mechanism of action has also been demonstrated for potassium channels such as K_V_1.5 (Mia et al., 2012), K_V_1.7 (Andersen et al., 2012), and K_V_7.1 (Alzamora et al., 2010). AMPK activation could similarly enhance NEDD4-2 function and favor sodium channel internalization in sensory neurons. In a previous study, researchers showed that downregulation of NEDD4-2 is associated with an increase in total sodium current (*I*_Na_) and voltage-gated sodium channel 1.7 (Na_V_1.7) currents in the SNI model of neuropathic pain. Restoring normal NEDD4-2 expression after SNI prevented full development of hypersensitivity (Laedermann et al., 2013). This leaves open the question as to whether metformin can pharmacologically modulate NEDD4-2 function to decrease sodium current expression, in particular Na_V_1.7, which in turn might explain its effect in various experimental models of chronic pain.

In this study, we test the hypothesis that metformin inhibits *I*_Na_ and Na_V_1.7 currents by decreasing their expression at the plasma cell membrane via NEDD4-2. In a heterologous system, we demonstrate that cotransfection of Na_V_1.7 and NEDD4-2 results in a significant decrease in the expression of the functional form of this channel coupled with lower current densities on metformin treatment. In *Nedd4-2^fl/fl^* mice, we show that metformin reduces both current densities and membrane expression of Na_V_1.7 compared with the *SNS-Nedd4L*^−/−^ knock-out animals. Furthermore, the excitability of dorsal root ganglion (DRG) neurons decreases after metformin treatment, there are altered parameters of single action potentials (APs), and there is hyperpolarization of the resting membrane potential (RMP). Our results show that metformin reduces both the expression and current densities of Na_V_1.7 in a NEDD4-2-dependent manner, whereas neuronal excitability decreases in both *Nedd4-2^fl/fl^* and *SNS-Nedd4L*^−/−^ knock-out animals. These findings provide new avenues for research investigating the effect of metformin on neuronal excitability independent of NEDD4-2.

## Materials and Methods

### Study approval

All experiments involving animals were performed according to the regulations of the University of Lausanne animal care committee. This investigation conformed to the Swiss Federal Laws on Animal welfare, the *Guide for the Care and Use of Laboratory Animals* (National Institutes of Health, 1996), and to the International Association for the Study of Pain guidelines for the use of animal in research (Zimmermann, 1983).

### Animals

Animals were grouped housed, in a standard environment (litter, paper roll, and tissue), with free access to food and water, and a 12 h photoperiod. The transgenic mouse line *SNS-Nedd4L*^−/−^ and their control littermates *Nedd4L^fl/fl^* were used. Briefly, the floxed *Nedd4L* mouse line (Nedd4l^tm1.1Blyg^; catalog #3846430, MGI; RRID:MGI:3846430 provided by Prof. O. Staub, University of Lausanne, Lausanne, Switzerland) was crossed with the *SNS-Cre* line [Tg(Scn10a-cre)1Rkun; catalog #3042874, MGI; RRID:MGI:3042874; provided by R. Kuner, University of Heidelberg, Heidelberg, Germany) expressing the *Cre* recombinase under the promoter of Na_V_1.8 (*SNS*). This breeding leads to a conditional knockout of NEDD4-2 in sensory Na_V_1.8-positive DRG neurons. Five-to-8-week-old animals were used for experiments. Males and females were used for the experiments.

### Cell culture and transfection

Human embryonic kidney 293 (HEK293) cells (catalog #12022001, European Collection of Authenticated Cell Cultures; RRID:CVCL_0063) were cultured in DMEM (catalog #21885–025, Thermo Fisher Scientific) supplemented with 10% heat-inactivated fetal bovine serum (FBS; catalog #10082, Thermo Fisher Scientific) and 1% penicillin–streptomycin (P/S; catalog #P0781, Sigma-Aldrich), at 37°C in a 5% CO_2_ incubator. For patch-clamp experiments, in a T25 culture flask (catalog #353108, Corning), the cells were seeded at a final density of 2 × 10^6^ cells and transfected using the Ca^2+^-phosphate method with different conditions, as follows: 1 μg of Na_V_1.7 cDNA concomitantly with 0.3 μg of NEDD4-2 (low NEDD4-2 condition; ratio, 1:0.3) plus 0.7 μg of empty vector, 1 μg of NEDD4-2 (high NEDD4-2 condition; ratio, 1:1) alone, or 1 μg of empty vector alone (no NEDD4-2 condition). In all cases, 0.1 μg of pIRES-AcGFP1 (catalog #632435, Takara Bio) was added and used as a reporter gene. For biotinylation assays, HEK293 cells were cultured in a P100 dish (catalog #353003, Corning) at a final density of 9 × 10^6^ cells and transiently cotransfected with 6 μg of Na_V_1.7, as well as 1.8 μg of NEDD4-2 plus 4.2 μg of empty vector (low NEDD4-2 condition), 6 μg of NEDD4-2 (high Nedd4-2 condition), or 6 μg of empty vector (no NEDD4-2 condition), mixed with 30 μl of JetPEI (catalog #101-10N, Polyplus Transfection) and 250 μl of 150 mm NaCl (catalog #702–50, Polyplus Transfection). The QuikChange Mutagenesis Kit (catalog #200519, Agilent Technologies Stratagene) was used to generate NEDD4-2CS mutants by changing Cys801 into a Ser. The cells were used in patch-clamp or biochemical experiments 48 h post-transfection.

### Primary neuronal culture

Mice were killed with cervical dislocation/lethal injection of pentobarbital (50 mg/kg). For electrophysiological recordings, L4 and L5 DRGs were dissected and collected in oxygenated complete saline solution (CSS; composition: 137 mm NaCl, 5.3 mm KCl, MgCl_2_·, 6H_2_O, 25 mm sorbitol, 10 mm HEPES, and 3 mm CaCl_2_; pH adjusted to 7.2 with NaOH). DRGs were then harvested and digested in 5 ml of solution containing the following: liberase blendzyme TH (catalog #5401151001, Roche) at a concentration of 0.5 U/DRG, 12 μm EDTA (catalog #E5134, Sigma-Aldrich) in oxygenated CSS for 20 min at 37°C. Neurons were further digested with Liberase blendzyme TM (CSS #5401127001, Roche) in 5 ml of solution (0.5 U/DRG, 12 μm EDTA in 5 ml CSS) plus 30 U/ml papain (catalog #P3125, Sigma-Aldrich) for 10 min. Then, neurons were suspended in 1 ml of DRG medium containing DMEM/F12 (catalog #21331–020, Thermo Fisher Scientific) with 10% FBS and 1% P/S, supplemented with 1.5 mg of trypsin inhibitor (catalog #T6522, Sigma-Aldrich) and 1.5 mg of purified bovine serum albumin (BSA; catalog #A9647, Sigma-Aldrich). Mechanical dissociation (12 strokes) was performed to triturate the DRGs gently. Finally, isolated neurons were plated on 12 mm coverslips (bovine serum albumin #631–1577, VWR) coated with 0.1 mg/ml poly-d-lysine (bovine serum albumin #P7886, Sigma-Aldrich). Neurons were only recorded at 12 h to prevent neurite outgrowth that degrades the space clamp. For cell surface biotinylation experiments, L2–L6 DRGs were dissected from each side of the spinal cord and pooled to obtain 10 DRGs per sample. The dissociated neurons were split in two, for the control and the metformin conditions; plated on six-well plates (catalog #353224, Corning); and kept in culture for 6 d before metformin treatment.

### Cell surface biotinylation assay

HEK293 cells transiently cotransfected or DRG neurons in culture were washed with cold 1× PBS, pH 7.4 (catalog #10010, Thermo Fisher Scientific) and then treated with 0.5 mg/ml EZ-link Sulfo-NHS-SS-Biotin (catalog #21331, Thermo Fisher Scientific) in cold 1× PBS for 30 min at 4°C. The cells were then washed three times with 200 mm glycine (catalog #A1067, AppliChem) in cold 1× PBS to inactivate biotin, and twice with cold 1× PBS to remove excess biotin. The cells were scraped and lysed with 1× lysis buffer that contained 50 mm HEPES, pH 7.4, 100 mm NaCl, 1 mm EGTA, pH 8, 10% glycerol, 1% Triton X-100, 10 mm *N*-ethylmaleimide, complete protease inhibitor cocktail (catalog #11697498001, Roche), and phosphatase inhibitor cocktail (catalog #04906837001, Roche) for 1 h at 4°C on a wheel. Whole-cell lysates were centrifuged at 16,000 × *g* for 15 min at 4°C. The protein concentration of supernatants was measured by using a Bradford-based assay (catalog #500–0006, BIO-RAD). Subsequently, 2 mg (500 μg for neurons) of the supernatant was incubated with 30 μl of Streptavidin Sepharose High Performance Beads (catalog #17–5113-01, GE Healthcare) for 2 h at 4°C on the wheel, and the remaining supernatants were kept as input fractions (S0 fractions). The beads were subsequently washed five times with 1× lysis buffer plus 1 mm phenylmethanesulfonyl fluoride (catalog #P7626, Sigma-Aldrich) with centrifugation steps (1000 × *g* for 2 min at 4°C) between washes (the supernatant was discarded after each centrifugation). Elution was performed with 40 μl of 5× sample buffer containing 1.5 m sucrose, 10% SDS (catalog #L4390, Sigma-Aldrich), 12.5 mm EDTA, 0.3 m Tris, pH 8.8, 0.25% bromophenol blue, and 150 mm dithiothreitol (catalog #A1101, AppliChem), at 37°C for 1 h with gentle shaking. A last centrifugation at 2500 × *g* for 1 min was performed to separate the beads from the supernatant fractions. These biotinylated fractions (S2 fractions) were analyzed by Western blot for Na_V_1.7 expression at the cell surface. The input fractions were diluted 1:5 in 5× sample buffer, incubated at 37°C for 30 min, and used to evaluate the total expression of Na_V_1.7 and the other proteins of interest by Western blot.

### Western blot

Proteins were separated on 7.5% acrylamide gels (catalog #10681, SERVA Electrophoresis) by using SDS-PAGE and then transferred to polyvinylidene fluoride membranes (catalog #162–0177, BIO-RAD). Subsequently, the membranes were immunoblotted with the following antibodies: anti-AMPK (1:1000 in BSA; rabbit polyclonal; catalog # 2532, Cell Signaling Technology; RRID:AB_330331); anti-phospho-AMPK (1:1000 in BSA; rabbit polyclonal; catalog #2531, Cell Signaling Technology; RRID:AB_330330); anti-Na_V_1.7 (1:500 in milk; mouse monoclonal clone N68/6; catalog #75–103, Antibodies Incorporated; RRID:AB_2184355); anti-NEDD4-2 (1:1000 in milk; rabbit polyclonal; catalog #ab46521, Abcam; RRID:AB_2149325); anti-Ser-328 phospho-NEDD4-2 (1:500 in milk; rabbit polyclonal; catalog #ab95399, Abcam; RRID:AB_10679598; provided by O. Staub, Lausanne University, Lausanne, Switzerland); and anti-α-tubulin used as a reference protein (1:20,000 in milk; mouse monoclonal clone B-5–1-2; catalog #T5168, Sigma-Aldrich; RRID:AB_477579). We used secondary peroxidase-linked goat anti-rabbit or goat anti-mouse IgG [1:10,000; catalog #P0448, Agilent (RRID:AB_2617138); and catalog #P0447, Agilent (RRID:AB_2617137)] and SuperSignal West Dura Extended Duration Substrate (catalog #34 075, Thermo Fisher Scientific) for detection. Chemiluminescence was detected by using an imaging system (model LAS-4000 Imaging System; ImageQuant, GE FujiFilm; RRID:SCR_014246) coupled with an integrated CCD camera. Protein quantification was performed using ImageJ software (Fiji; RRID:SCR_002285; Schindelin et al., 2012). The quantified signals for the proteins of interest were normalized to reference protein signals.

### Solutions

Transfected HEK293 cells and DRG neurons in culture were treated with 20 mm metformin (catalog #LKT-M2076-G025, LKT Labs) solubilized in their respective culture media, for 12 h, before the assessment of electrophysiological recordings or cell surface biotinylation assays. The concentration of metformin applied was the optimum for cell survival according to various dose–response assays (data not shown). Control groups were incubated with their respective culture media only.

For HEK293 cells, whole-cell voltage-clamp recordings were conducted by using an internal solution containing 60 mm CsCl, 70 mm aspartic acid, 11 mm EGTA, 1 mm MgCl_2_, 1 mm CaCl_2_, 10 mm HEPES, and 5 mm Na_2_-ATP, at pH 7.2 adjusted with CsOH, and an external solution containing 130 mm NaCl, 2 mm CaCl_2_, 1.2 mm MgCl_2_, 5 mm CsCl, 10 mm HEPES, and 5 mm glucose, at pH 7.4 adjusted with CsOH). For DRGs, the internal solution contained 140 mm CsF, 10 mm NaCl, 2 mm MgCl_2_, 0.1 mm CaCl_2_, 1.1 mm EGTA, and 10 mm HEPES, at pH 7.2 adjusted with CsOH and the osmolarity adjusted to 310 mOsm with glucose. The extracellular solution contained 30 mm NaCl, 20 mm TEA-Cl, 90 mm choline-Cl, 3 mm KCl, 1 mm CaCl_2_, 1 mm MgCl_2_, 10 mm HEPES, 10 mm glucose, and 0.1 mm CdCl, at pH 7.3 adjusted using Tris base and the osmolarity adjusted to 320 mOsm with glucose (Cummins et al., 2009). The current-clamp recordings were conducted by using an internal solution containing 140 mm KCl, 0.5 mm EGTA, 3 mm Mg-ATP, and 5 mm HEPES, at pH 7.3 adjusted with KOH. The extracellular solution contained 140 mm NaCl, 3 mm KCl, 2 mm CaCl_2_, 2 mm MgCl_2_, and 10 mm HEPES, at pH 7.3 adjusted using NaOH and the osmolarity adjusted to 320 mOsm with glucose. See Extended Data Table 1-1 for the full list of products.

**Table 1:** Biophysical properties of Na_V_1.7-mediated currents in HEK293 cells, transfected with different NEDD4-2 plasmids, with (+) or without (–) metformin treatment.

	Na_V_1.7 alone		Na_V_1.7 + Low [NEDD4-2]		Na_V_1.7 + NEDD4-2CS	
Metformin	–	+	–	+	–	+
SSA						
V_1/2_ (mV)	−8.1 ± 0.7	−6.3 ± 1.1	−6.7 ± 1.1	−5.4 ± 1.4	−9.3 ± 0.6	−9.1 ± 0.9
Slope	6.5 ± 0.2	7.1 ± 0.3	6.0 ± 0.3	**7.2 ± 0.3	7.1 ± 0.3	7.1 ± 0.2
*n*	30	17	16	11	12	11
SSI						
V_1/2_ (mV)	−61.3 ± 1.1	−61.5 ± 1.3	−58.0 ± 1.5	−58.9 ± 1.8	−65.8 ± 1.2	−65.2 ± 1.3
Slope	7.45 ± 0.4	*9.01 ± 0.6	8.82 ± 0.7	7.75 ± 0.6	7.72 ± 0.5	7.1 ± 0.3
*n*	30	16	15	14	12	12

The three groups correspond to HEK293 cells transfected only with Na_V_1.7, with Na_V_1.7 and NEDD4-2 in low concentration (Na_V_1.7 cDNA/NEDD4-2 cDNA ratio 1:0.3), or with Na_V_1.7 and an inactivated NEDD4-2, mutated on the catalytic site. The *V*_1/2_ (mV) is the voltage at which half of the available channels are activated or inactivated based on their steady-state protocol. Data are expressed as the mean ± SEM. Statistical analysis was performed using an unpaired *t* test with Welch’s correction (Extended Data Table 1-2). Detailed references for the resources used are presented in Extended Data Table 1-1.

*p < 0.05, **p < 0.01, ***p < 0.001. The bold values correspond to the values, which shows a statistically significant difference.

### Electrophysiology

Data were acquired with a Multiclamp 700B Microelectrode Amplifier and pClamp 10 software (Molecular Devices; RRID:SCR_011323) or with an EPC-9 Amplifier and Patchmaster software (HEKA Electronics; RRID:SCR_000034). Data were analyzed with KaleidaGraph 4.03 (Synergy Software; RRID:SCR_014980) and GraphPad Prism 7 (GraphPad Software; RRID:SCR_002798). Low-pass filtering was set to 5.0 kHz. Resistance of the borosilicate pipettes (catalog #BF150-86–7.5, Sutter Instrument) was 1.5–3 MΩ. After opening, HEK293 cells were kept at −70 mV for at least 2 min to dialyze and equilibrate the cell. DRG neurons were kept at −60 mV for 5 min to dialyze the cell and to allow Na_V_1.8 voltage dependencies to stabilize and inactivate Na_V_1.9 currents. Cells were then clamped at −80 mV for 2 min before starting the recordings. The *I*_Na_ current densities (pA/pF) were obtained by dividing the peak *I*_Na_ by the cell capacitance.

The current-clamp recordings were made on cultured primary neurons from *Nedd4L^fl/fl^* and *SNS-Nedd4L*^−/−^ animals. Recordings were made following a 500 ms ramp current ranging from 100 to 500 pA, and the APs were counted and represented at each ramp. Small step pulses of 5 ms with 25 pA increments were done, and the first AP triggered was analyzed for different parameters. Peak amplitude represents the maximum amplitude of the AP measured from the RMP. The maximum rise time is the time from the start of the stimulation until the maximum AP amplitude was reached. The baseline is the voltage amplitude starting from the AP threshold until the start of the AP overshoot, and the rheobase is the minimum current needed to elicit an AP.

The voltage-dependent steady-state activation (SSA) curves were determined from *I–V* curves, where the Na^+^ current was evoked from a holding potential of −100 mV to test pulses of 100 ms ranging from −80 to +40 mV in increments of 5 mV. For DRG neuron recordings, each step was preceded by a 3 s prepulse at −120 mV. The leakage current was subtracted by using the P/4 procedure in Clampex software. The sodium channel conductance was calculated following the formula *G*_Na_ = *I*_Na_*/*(*V_m_* – *V*_rev_), where *I*_Na_ is the peak current amplitude, *V_m_* is the current potential, and *V*_rev_ is the reversal potential for the current determined by a linear fit. The activation curve for each individual cell was fitted with a Boltzmann equation with the formula *G*_Na_ = *G*_max_*/*(1 + exp [(*V*_1/2_ – *V_m_*)/*k*]), where *G*_max_ is the maximum conductance, *V*_1/2_ is the potential at which half of the sodium channels are activated, *V_m_* is the current potential, and *k* is the slope factor.

On the other hand, the voltage-dependent steady-state inactivation (SSI) curves were measured from a holding potential of −100 mV using 500 ms prepulses ranging from −130 to +5 mV, in increments of 5 mV, followed by a test pulse to 0 mV. The inactivation curves were fitted with the Boltzmann relationship as follows: *I*_Na_ = *I*_max_/(1 + exp [(*V*_1/2_ – *V_m_*)/*k*]), where *I*_max_ is the maximum value for the sodium current, *V*_1/2_ is the potential at which *I*_Na_ is half-inactivated, *V_m_* is the membrane potential achieved using a prepulse step, and *k* is the slope factor. Five nanomolar Protoxin II (ProTxII; catalog #P0033, Sigma-Aldrich) was used to isolate Na_V_1.7-mediated currents from DRG primary culture (Schmalhofer et al., 2008).

### Statistical analyses

For statistical analysis, we used GraphPad Prism and R software. Data are presented as a box plot with whiskers extending from the 10th to 90th percentile (minimum to maximum, including every individual point), or the mean ± SEM. Unpaired *t* test with Welch’s correction was used to compare the means between two groups, and two-way repeated-measures ANOVA, followed by a *post hoc* analysis using a Sidak’s multiple-comparison test, was used to compare the effect of metformin treatment in different conditions; *p* < 0.05 was considered to be significant. The statistical tests, *t*, *F*, and *p* values, and the effect size calculated with Cohen’s factors are provided in Extended Data Table 1-2.

**Table 2:** Biophysical properties of total Na_V_ and Na_V_1.7-mediated currents in DRG neurons from *Nedd4L^fl/fl^* or *SNS-Nedd4L*^−/−^ mice, with (+) or without (–) metformin treatment

	*Nedd4-2^fl^*^/^*^fl^* mice				*SNS-Nedd4-2*^–/–^ mice			
	Na_V_ total		Na_V_1.7		Na_V_ total		Na_V_1.7	
Metformin	–	+	–	+	–	+	–	+
SSA								
V_1/2_ (mV)	−37.1 ± 2.3	−35.6 ± 3.4	−42.8 ± 3.2	−40.4 ± 4.7	−37.0 ± 2.7	−39.9 ± 4.8	−36.5 ± 14.0	−43.2 ± 4.9
Slope	6.5 ± 0.5	6.6 ± 0.6	5.9 ± 1.0	5.2 ± 1.2	6.2 ± 0.4	**8.2 ± 0.4	7.0 ± 1.5	5.9 ± 0.6
*n*	15	10	6	5	12	8	3	6

The SSA was measured on total *I*_Na_ current density or Na_V_1.7-mediated currents for each genotype and treatment condition. The *V*_1/2_ (mV) is the voltage at which half of the available channels are activated with the SSA protocol. Data are expressed as the mean ± SEM. Statistical analysis was performed using an unpaired *t* test with Welch’s correction (Extended Data Table 1-2). Detailed references for the resources used are presented in Extended Data Table 1-1.

*p < 0.05, **p < 0.01, ***p < 0.001. The bold values correspond to the values, which shows a statistically significant difference.

## Results

### *In vitro*, the effect of metformin on the Na_V_1.7 current density depends on the presence of NEDD4-2

**Figure 1.**
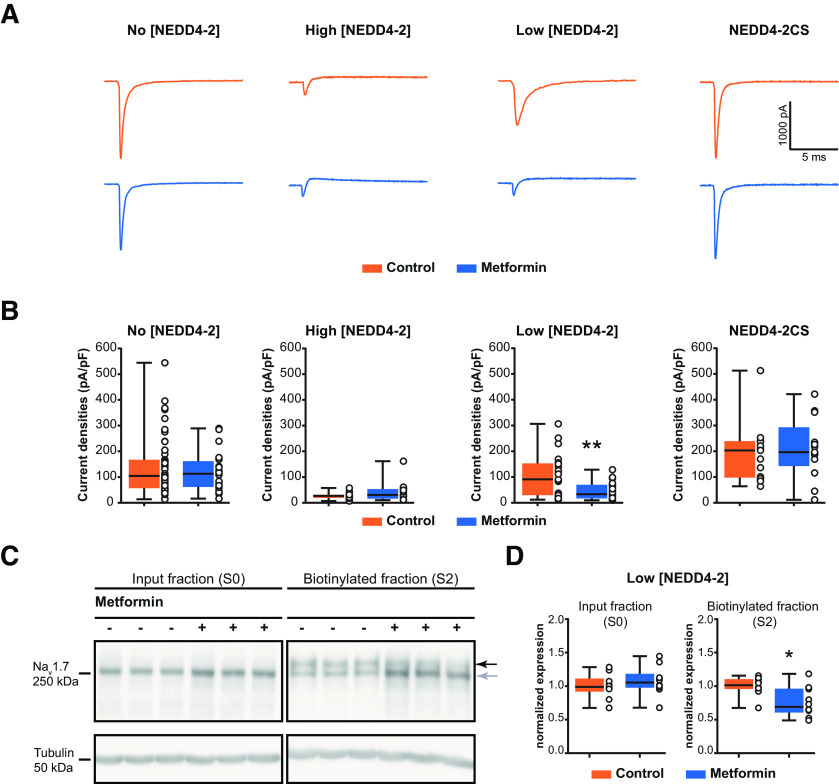
The effect of metformin on the Na_V_1.7 peak current depends on the presence of NEDD4-2 in HEK293 cells. ***A***, Representative traces of Na_V_1.7 recordings, at the maximum peak current, from Na_V_1.7-transfected HEK293 cells, cotransfected with different concentrations of NEDD4-2 plasmid. These data are supported by further data presenting the multiple traces, obtained at each step of the protocol, in Extended Data Fig. 1-2. Cells were incubated with culture medium for control conditions (top) or 20 mm metformin (bottom) over 12 h. High [NEDD4-2] (ratio 1:1), Low [NEDD4-2] (ratio 1:0.3), and NEDD4-2CS (mutated form of NEDD4-2 with inactivated catalytic site) conditions are presented. ***B***, Bar graphs presenting the Na_V_1.7 peak current in the four conditions with control medium or 20 mm metformin (*n* = 12–47 cells recorded/group). ***C***, Representative blots of surface biotinylation of HEK293 cells cotransfected with Na_V_1.7 and Low [NEDD4-2]. The upper band corresponds to the fully glycosylated form of Na_V_1.7, while the lower band corresponds to the core glycosylated form. ***D***, Respective quantification of Na_V_1.7 expression in total input fraction (S0) and biotinylated fraction (S2), with control medium or metformin treatment (*n* = 10/condition). Values were normalized to tubulin expression. These data are supported by further data presenting Na_V_1.7 expression, with other concentration of NEDD4-2, in Extended Data Fig. 1-1. Data are represented as a box plot with whiskers extending from the 10th to the 90th percentile, and each dot represents one recorded cell (***A***) or one sample (***D***). Statistical analysis was performed using an unpaired *t* test with Welch’s correction for both patch-clamp and Western blot datasets (Extended Data Table 1-2). *p < 0.05, **p < 0.01, ***p < 0.001. Detailed references for the resources used are presented in Extended Data Table 1-1.

We performed whole-cell patch-clamp experiments in HEK293 cells cotransfected with Na_V_1.7 and different concentrations of NEDD4-2 plasmids. Figure 1, *A* and *B*, shows, respectively, representative traces of the Na_V_1.7 current and the quantification of the maximum Na_V_1.7 current density, from a holding potential of −100 mV to test pulses of 100 ms ranging from −80 to +40 mV in increments of 5 mV, in the different NEDD4-2 concentration conditions (Extended Data Fig. 1-2).

10.1523/ENEURO.0409-21.2022.f1-1 Figure 1-1 Supplemental data figure supporting Figure 1. Effect of metformin on Na_V_1.7 expression in HEK293 cells, depending on NEDD4-2 expression levels. ***A***, Representative blots of the total input fraction (S0) or surface biotinylation (S2) of HEK293 cells cotransfected with Na_V_1.7 and No [NEDD4-2], treated with medium or metformin. ***B***, Respective quantification of Na_V_1.7 expression in S0 (left) and S2 (right), with control medium or metformin treatment (*n* = 6/condition). ***C***, Representative blots of the S0 or S2 of HEK293 cells cotransfected with Na_V_1.7 and High [NEDD4-2], treated with control medium or metformin. ***D***, Respective quantification of Na_V_1.7 expression in S0 (left) and S2 (right), with control medium or metformin treatment (*n* = 6/condition). Values were normalized to tubulin expression. Data are represented as a box plot with whiskers extending from the 10th to the 90th percentile; each dot represents one sample. Statistical analysis was performed using an unpaired *t* test with Welch’s correction (Extended Data Table 1-2). *p < 0.05, **p < 0.01, ***p < 0.001. Detailed references for the resources used are presented in Extended Data Table 1-1. Download Figure 1-1, EPS file.

**Figure 2.**
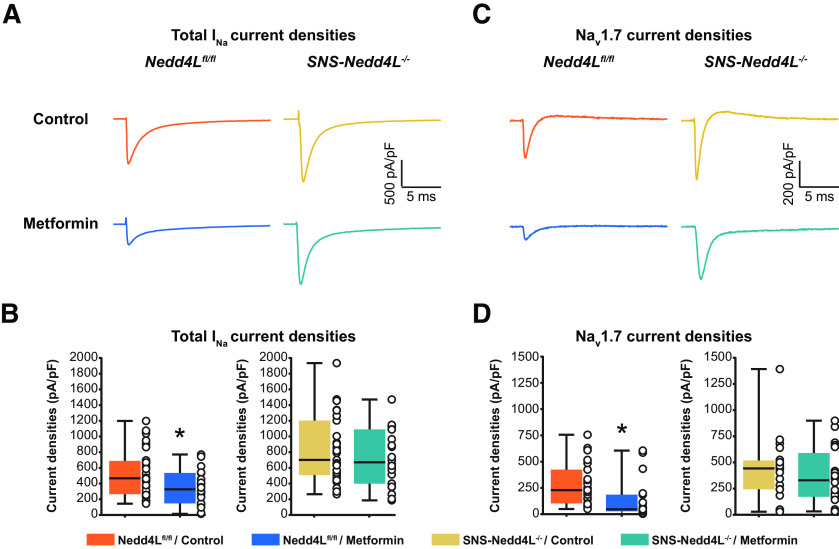
Metformin reduced the total *I*_Na_ and Na_V_1.7 current densities in DRG neurons in a NEDD4-2-dependent manner. DRG neuronal primary cultures were incubated for 12 h with culture medium or 20 mm metformin. ***A***, Representative traces of total *I*_Na_, at the maximum peak current, recorded with an *I–V* protocol in DRG neurons from *Nedd4-L^fl/fl^* mice or *SNS-Nedd4-L*^−/−^ mice. The traces were normalized to the capacitance of the recorded cell and expressed in pA/pF over time. These data are supported by further data presenting the multiple traces, obtained at each step of the protocol, in Extended Data Figure 2-1. ***B***, Quantified total *I*_Na_ current density in the four conditions (*n* = 22–28 cells recorded/condition). ***C***, Representative traces of Na_V_1.7 current density, at the maximum peak current, recorded with a *I–V* protocol. Na_V_1.7 currents were isolated from the total *I*_Na_ by acute application of ProTxII at 5 nm during the recordings. The traces were normalized to the capacitance of the recorded cell and expressed in pA/pF over time. ***D***, Quantified Na_V_1.7 current density in the four similar conditions (*n* = 19–23 cells recorded/condition). Data are represented as a box plot with whiskers extending from the 10th to the 90th percentile (minimum to maximum), with each dot representing a single recorded cell. Statistical analysis was performed using an unpaired *t* test with Welch’s correction (Extended Data Table 1-2). *p < 0.05, **p < 0.01, ***p < 0.001. Detailed references for the resources used are presented in Extended Data Table 1-1.

10.1523/ENEURO.0409-21.2022.f1-2 Figure 1-2 Supplemental data figure supporting the Figure 1. Representative example traces of Na_V_1.7 currents, at each step of the protocol. The voltage-clamp protocol used is represented below the traces. It consisted in evoked sodium currents, at a holding potential of –100 mV, with pulses of 100 ms ranging from –80 to +40 mV in increments of 5 mV. Recordings from transfected HEK293 cells, cotransfected with Na_V_1.7 and different concentrations of NEDD4-2 plasmid. Cells were incubated with culture medium for control conditions (top) or 20 mm metformin (bottom) during 12 h. High [NEDD4-2] (ratio 1:1), Low [NEDD4-2] (ratio 1:0.3), and NEDD4-2CS (mutated form of NEDD4-2 with inactivated catalytic site) conditions are presented. Detailed references for the resources used are presented in Extended Data Table 1-1. Download Figure 1-2, EPS file.

Treatment with 20 mm metformin for 12 h did not change Na_V_1.7 peak current in HEK293 cells transfected only with Na_V_1.7 (No [NEDD4-2], control vs metformin: *p* = 0.3907, *t*_(51.07)_ = 0.8657, *n* = 20 or 47/group). When cotransfected with NEDD4-2 at a high concentration (Na_V_1.7 cDNA/NEDD4-2 cDNA was 1:1 ratio, High [NEDD4-2]), the reduction in Na_V_1.7, because of the ubiquitin ligase, probably reaches its maximum effect, masking the effect of metformin treatment on Na_V_1.7 peak current (High [NEDD4-2], control vs metformin: *p* = 0.2456, *t*_(13.2)_ = 1.215, *n* = 12/group). We thus used a lower [NEDD4-2] concentration (Na_V_1.7 cDNA/NEDD4-2 cDNA ratio was 1:0.3, termed Low [NEDD4-2]), and observed that the metformin treatment led to a significant decrease in Na_V_1.7 peak current (Low [NEDD4-2], control vs metformin: *p* = 0.0034, *t*_(26.57)_ = 3.221, *n* = 21/group). Moreover, in HEK293 cells cotransfected with a mutated NEDD4-2 (NEDD4-2CS), in which the catalytic site is inactivated, the metformin treatment did not change Na_V_ peak currents (NEDD4-2CS, control vs metformin: *p* = 0.743, *t*_(24)_ = 0.3316, *n* = 13/group). Metformin did not modify the *V*_1/2_ of activation of Na_V_1.7 in the Low [NEDD4-2] condition (control vs metformin: *p* = 0.4506, *t*_(21.1)_ = −0.77, *n* = 11–16/group). Nevertheless, the slope of Na_V_1.7 activation appeared significantly faster on metformin treatment (control vs metformin: *p* = 0.005, *t*_(24.3)_ = −3.08, *n* = 11–16/group; Table 1).

Then, we investigated whether Na_V_1.7 cell surface expression was changed by metformin. Figure 1*C* shows representative blots of Na_V_1.7 in the total input fraction or in the cell surface biotinylation experiment. While the total amount of Na_V_1.7 was unchanged (input fraction, control vs metformin: *p* = 0.3871, *t*_(17.47)_ = 0.8871, *n* = 10/group), the fully glycosylated form of Na_V_1.7 (Fig. 1*C*, top band) was significantly decreased in the plasma membrane fraction (biotinylated fraction, control vs metformin: *p* = 0.0104, *t*_(14.99)_ = 2.926, *n* = 10/group; Fig. 1*D*). Metformin had no effect on Na_V_1.7 expression in either the absence of NEDD4.2 (control vs metformin: *p* = 0.068, *t*_(8.144)_ = 2.104, *n* = 6/group) or on high concentration of NEDD4.2 (control vs metformin: *p* = 0.5753, *t*_(9.524)_ = 0.5801, *n* = 6/group) cotransfected with Na_V_1.7 (Extended Data Fig. 1-1).

### Metformin downregulates total *I*_Na_ and Na_V_1.7 current densities in DRG neurons via NEDD4-2

We used freshly dissociated L4 and L5 mouse small DRG neurons with a membrane capacitance <30 pF, considered to be nociceptive neurons (Lopez-Santiago et al., 2006), and performed whole-cell patch-clamp recordings of total *I*_Na_ and Na_V_1.7 currents (Fig. 2*A*,*C*, Extended Data Fig. 2-1). From total *I*_Na_ currents, we pharmacologically isolated Na_V_1.7 current by using 5 nm ProTxII, a concentration at which the toxin blocks Na_V_1.7 selectively (Schmalhofer et al., 2008). To investigate the contribution of NEDD4-2 expression in the metformin-mediated downregulation of the Na_V_1.7 current, we used a DRG neuron-specific NEDD4-2 knock-out mouse line (*SNS-Nedd4L*^−/−^). Mice heterozygously expressing *Cre* recombinase under the control of the Na_V_1.8 promoter (*SNS-Cre*; Agarwal et al., 2004) were crossed with homozygous mice carrying the Nedd4-2 floxed allele (*Nedd4L^fl/fl^*; Shi et al., 2008). In the *Nedd4L^fl/fl^* control mice, the total *I*_Na_ was significantly decreased by the metformin treatment (total *I*_Na_ *Nedd4L^fl/fl^*, control vs metformin: *p* = 0.0139, *t*_(47.72)_ = 2.554, *n* = 22 or 28/group; Fig. 2*B*, left). Conversely, the total *I*_Na_ current density was not significantly changed in the DRG neurons from the *SNS-Nedd4L*^−/−^ mice (total *I*_Na_ *SNS-Nedd4L*^−/−^, control vs metformin: *p* = 0.3637, *t*_(48.92)_ = 0.9169, *n* = 23 or 27/group; Fig. 2*B*, right). Similarly, a >50% decrease in Na_V_1.7 current density was observed on metformin incubation in the control mice (Na_V_1.7 current density in *Nedd4L^fl/fl^*, control vs metformin: *p* = 0.0184, *t*_(39.09)_ = 2.461, *n* = 19 or 23/group; Fig. 2*D*, left), but not in the DRG neurons from the *SNS-Nedd4L*^−/−^ mice (Na_V_1.7 current density in *SNS-Nedd4L*^−/−^, control vs metformin: *p* = 0.4717, *t*_(37.18)_ = 0.7271, *n* = 20/group; Fig. 2*D*, right). Furthermore, similar to the results in HEK293 cells, metformin did not modify the *V*_1/2_ of activation of either total *I*_Na_ or Na_V_1.7 in DRG neurons from the *Nedd4L^fl/fl^* mice (Table 2).

10.1523/ENEURO.0409-21.2022.f2-1 Figure 2-1 Supplemental data figure supporting Figure 2. Representative example traces of total *I*_Na_ or Na_V_1.7 currents, at each step of the protocol, recorded from different DRG neurons, with or without metformin treatment. The voltage-clamp protocol used is represented below the traces. It consisted in evoked sodium currents, at a holding potential of –100 mV, with pulses of 100 ms ranging from –80 to +40 mV in increments of 5 mV. ***A***, Recordings of total *I*_Na_ from DRG neurons of *Nedd4-L^fl/fl^* mice or *SNS-Nedd4-L^–/–^* mice. The traces were normalized to the capacitance of the recorded cell and expressed in pA/pF over time. ***B***, Recordings of Na_V_1.7 current density from DRG neurons of *Nedd4-L^fl/fl^* mice or *SNS-Nedd4-L^–/–^* mice. Na_V_1.7 currents were isolated from the total *I*_Na_ by acute application of ProTxII at 5 nm during the recordings. The traces were normalized to the capacitance of the recorded cell and expressed in pA/pF over time. Detailed references for the resources used are presented in Extended Data Table 1-1. Download Figure 2-1, EPS file.

10.1523/ENEURO.0409-21.2022.tab1-1 Table 1-1 Supplemental data table of reagents, solutions, and RRID references, supporting all of the figures. RRID and source references of the resources used in the current study are noted. Download Table 1-1, XLSX file.

10.1523/ENEURO.0409-21.2022.tab1-2 Table 1-2 Supplemental data table of all statistical analysis, supporting all of the figures. Statistical table. Each row represents a dataset and the corresponding statistical tests, statistical values, *p* values, and Cohen’s effect size factor. Download Table 1-2, XLSX file.

### The reduction in currents parallels a downregulation of Na_V_1.7 cell surface expression in DRG neurons

**Figure 3.**
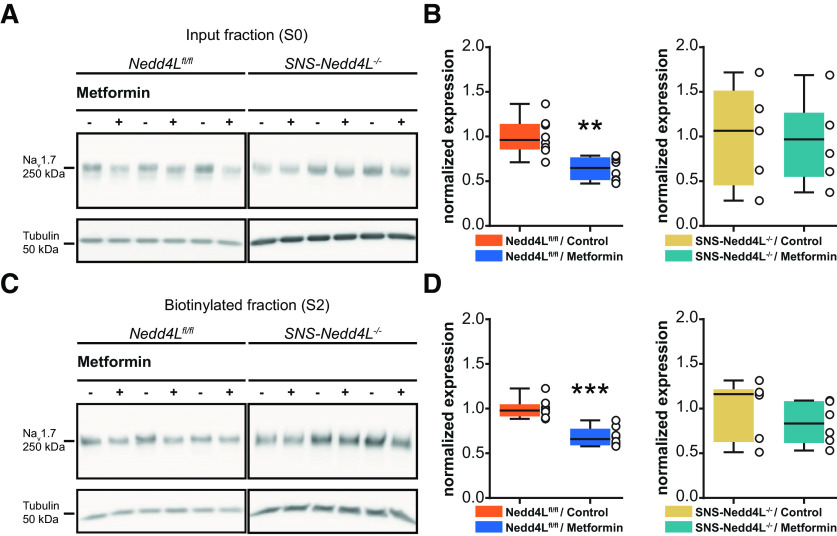
Effect of metformin on Na_V_1.7 expression in DRG neurons. Primary cultures of DRG were incubated for 12 h with medium alone or 20 mm metformin. ***A***, Representative blots of Na_V_1.7 expression in total input fraction (S0) on DRG neurons from *Nedd4L^fl/fl^* (left) or *SNS-Nedd4L*^−/−^ (right). ***B***, Quantification of Na_V_1.7 expression on the input fraction from DRG neurons of *Nedd4L^fl/fl^* (*n* = 8/treatment) or *SNS-Nedd4L*^−/−^ (*n* = 5–6/treatment), treated or not with metformin. ***C***, Representative blots of Na_V_1.7 expression in biotinylated fraction (S2) on DRG neurons from *Nedd4L^fl/fl^* (left) or *SNS-Nedd4L*^−/−^ (right). ***D***, Quantification of Na_V_1.7 expression on the biotinylated fraction from DRG neurons of *Nedd4L^fl/fl^* (*n* = 8/treatment) or *SNS-Nedd4L*^−/−^ (*n* = 6/treatment), treated or not with metformin. Values were normalized to tubulin expression. These data are supported by further data presenting AMPK and p-AMPK expression, with or without metformin treatment, in Extended Data Figure 3-1. Data are represented as a box plot with whiskers extending from the 10th to the 90th percentile (minimum to maximum), with each dot representing a single sample. Statistical analysis was performed using an unpaired *t* test with Welch’s correction (Extended Data Table 1-2). *p < 0.05, **p < 0.01, ***p < 0.001. Detailed references for the resources used are presented in Extended Data Table 1-1.

Metformin treatment led to a significant decrease in Na_V_1.7 expression in the total input fraction and in the biotinylated fraction of cultured DRG neurons from the control mice (input fraction *Nedd4L^fl/fl^*, control vs metformin: *p* = 0.0013, *t*_(11.79)_ = 4.205, *n* = 8/group; biotinylated fraction *Nedd4L^fl/fl^*, control vs metformin: *p* < 0.0001, *t*_(13.99)_ = 5.957, *n* = 8/group; Fig. 3*B*,*D*, left panels). By contrast, in the absence of NEDD4-2 in DRG neurons from the *SNS-Nedd4L*^−/−^ mice, no significant effect of metformin was observed in either the input or biotinylated fraction (input fraction *SNS-Nedd4L*^−/−^, control vs metformin: *p* = 0.8917, *t*_(7.794)_ = 0.1407, *n* = 5–6/group; biotinylated fraction *SNS-Nedd4L*^−/−^, control vs metformin: *p* = 0.3423, *t*_(9.066)_ = 1.002, *n* = 6/group; Fig. 3*B*,*D*, right panels).

10.1523/ENEURO.0409-21.2022.f3-1 Figure 3-1 Supplemental data figure supporting the Figure 3. Effect of metformin on AMPK and p-AMPK in HEK293 cells and DRG neurons. ***A***, Representative blots of AMPK and p-AMPK expression in HEK293 cells, treated with medium or 20 mm metformin. ***B***, Respective quantification of AMPK, p-AMPK expressions, and p-AMPK/AMPK ratio (*n* = 6/condition). ***C***, Representative blots of AMPK and p-AMPK expression in DRG neurons, treated with control medium or 20 mM metformin. ***D***, Respective quantification of AMPK, p-AMPK expressions, and p-AMPK/AMPK ratio (*n* = 6/condition). Values were normalized to tubulin expression. Data are represented as a box plot with whiskers extending from the 10th to the 90th percentile; each dot represents one sample. Statistical analysis was performed using an unpaired *t* test with Welch’s correction (Extended Data Table 1-2). *p < 0.05, **p < 0.01, ***p < 0.001. Detailed references for the resources used are presented in Extended Data Table 1-1. Download Figure 3-1, EPS file.

Among the kinases activated by metformin that regulate NEDD4.2 function (Melemedjian et al., 2011), we confirmed here that metformin increased AMPK phosphorylation in DRG neurons (Extended Data Fig. 3-1).

### Metformin decreases NEDD4-2 phosphorylation at Ser-328

**Figure 4.**
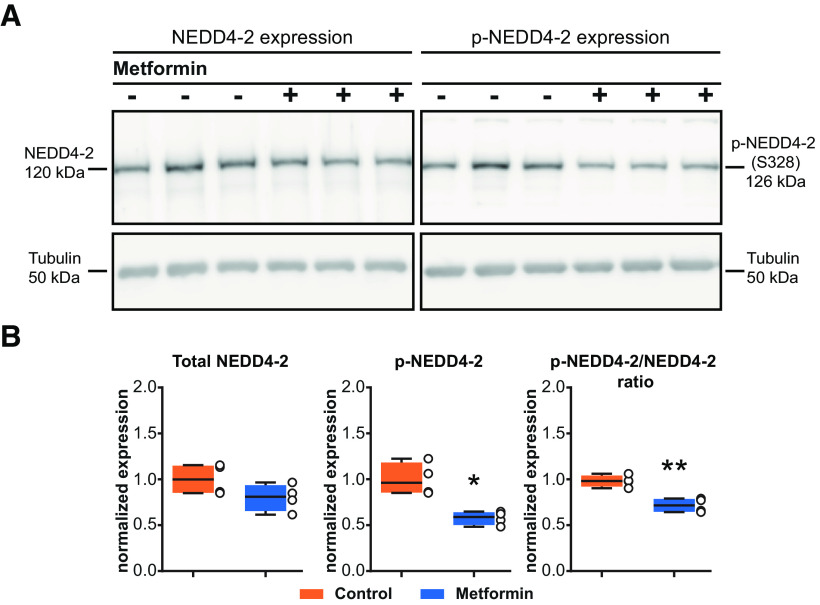
Metformin decreases NEDD4-2 phosphorylation. ***A***, Representative blots of the total NEDD4-2 expression (top) and the phosphorylated NEDD4-2 (at serine-328; bottom) in DRG neurons incubated with culture medium or with metformin. ***B***, Quantification of the metformin effect on NEDD4-2 expression (*n* = 4/treatment) and phosphorylated NEDD4-2 (Ser-328 residue; *n* = 4/treatment). For all datasets, values were normalized to tubulin expression, and the data are represented as a box plot with whiskers extending from the 10th to the 90th percentile (minimum to maximum), with each dot representing a single sample. Statistical analysis was performed using and unpaired *t* test with Welch’s correction (Extended Data Table 1-2). *p < 0.05, **p < 0.01, ***p < 0.001. Detailed references for the resources used are presented in Extended Data Table 1-1.

AMPK-mediated inhibition of ENaC currents has been suggested to be dependent on NEDD4-2 stabilization through phosphorylation at Ser-444 (Ho et al., 2018). Conversely, NEDD4-2–Ser-328 phosphorylation has been described to prevent the binding of NEDD4-2 on its targets (Flores et al., 2005). In the current study, metformin did not change NEDD4-2 expression (total NEDD4-2, control vs metformin: *p* = 0.1221, *t*_(5.916)_ = 1.803, *n* = 4/group), but significantly decreased NEDD4-2 phosphorylated (p) Ser-328 (p-NEDD4-2, control vs metformin: *p* = 0.0117, *t*_(4.005)_ = 4.391, *n* = 4/group; Fig. 4*A*,*B*). Consequently, we found a significant decrease in the p-NEDD4-2/NEDD4-2 ratio when treated with metformin compared with regular medium (p-NEDD4-2/NEDD4-2 ratio, control vs metformin: *p* = 0.0019, *t*_(5.847)_ = 5.328, *n* = 4/group). That change could allow the nonphosphorylated form of NEDD4-2 to bind more efficiently to its substrate (e.g., Na_V_1.7), promoting more internalization and reducing *I*_Na_ and the Na_V_1.7 current.

### Metformin reduces DRG neuron firing frequencies

**Figure 5.**
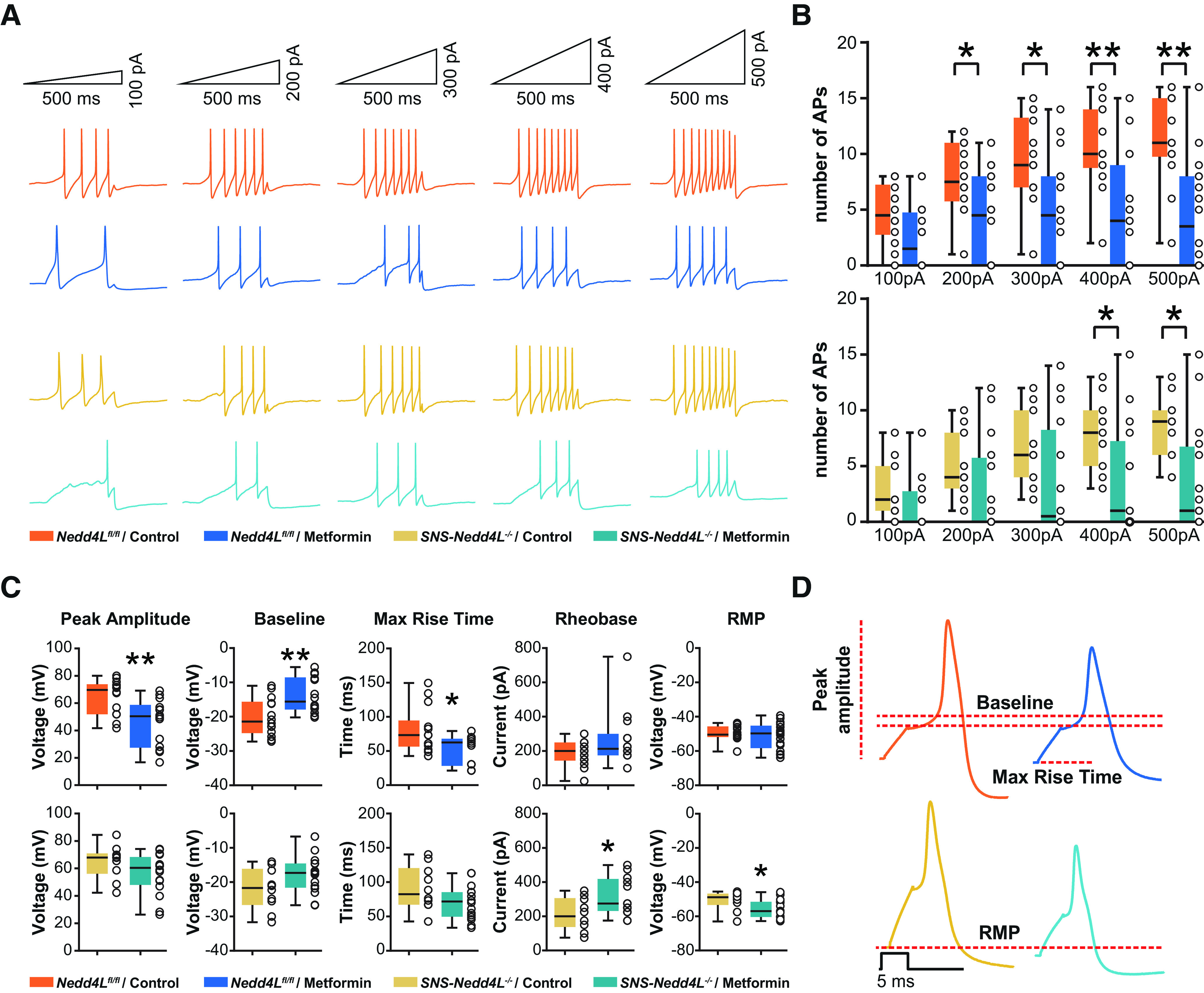
Current-clamp recordings of DRG neurons from *Nedd4L^fl/fl^* or *SNS-Nedd4L*^−/−^, treated with medium or 20 mm metformin. The recordings are acquired from at least three different patch-clamp sessions. ***A***, Representative traces of APs in different conditions elicited by ramp protocols of 500 ms (inset). ***B***, Box plots showing the analysis of APs number in *Nedd4L^fl/fl^* mice (top; *n* = 14–16/treatment) and *SNS-Nedd4L*^−/−^ mice (bottom; *n* = 11–16/treatment); data are represented as a box plot with whiskers extending from the 10th to the 90th percentile (minimum to maximum), with each dot representing a single recorded cell. Statistical analysis was performed using a two-way repeated-measures ANOVA, followed by Sidak’s multiple-comparisons *post hoc* test (*p < 0.05, **p < 0.01, ***p < 0.001; Extended Data Table 1-2). ***C***, Representative single AP and its parameters in different conditions, as illustrated by the color code. ***D***, Analysis of AP parameters, which are significantly changed on metformin treatment in *Nedd4L^fl/fl^* mice (*n* = 14 cells/treatment) or *SNS-Nedd4L*^−/−^ mice (*n* = 10–16 cells/treatment); data are represented as a box plot with whiskers extending from the 10th to the 90th percentile (minimum to maximum). These data are supported by further data presenting the AP characteristics, obtained from DRG neurons of *Nedd4L^fl/fl^* or *SNS-Nedd4L*^−/−^ mice treated with or without metformin, in Extended Data Figure 5-1. Statistical analysis was performed using an unpaired *t* test with Welch’s correction (Extended Data Table 1-2). *p < 0.05, **p < 0.01, ***p < 0.001. Detailed references for the resources used are presented in Extended Data Table 1-1.

A reduction in both total *I*_Na_ and Na_V_1.7 current densities after metformin treatment in the *Nedd4L^fl/fl^* mice suggests a reduction in neuronal excitability. We thus recorded DRG neurons in the current-clamp configuration (Fig. 5*A*). We found that metformin treatment decreased the number of APs in the *Nedd4L^fl/fl^* mice, at increased stimulation ramps (two-way repeated-measures ANOVA, current injection: *F*_(1.9,55.8)_ = 37.49, *p* < 0.0001; treatment: *F*_(1,28)_ = 27.3, *p* = 0.0019; interaction: *F*_(4,112)_ = 7.474, *p* < 0.0001, *n* = 14–16/treatment). *Post hoc* Sidak’s multiple-comparisons test revealed a significant treatment effect beginning at 200 pA (200 pA, *p* = 0.0413; 300 pA, *p* = 0.0247; 400 pA, *p* = 0.0057; 500 pA, *p* = 0.0021). There was also a reduction in the number of APs in DRG neurons from the *SNS-Nedd4L*^−/−^ mice (two-way repeated-measures ANOVA, current injection: *F*_(1.8,44.5)_ = 43.16, *p* < 0.0001; treatment: *F*_(1,125)_ = 4.6, *p* = 0.0419; interaction: *F*_(4,100)_ = 10.77, *p* < 0.0001, *n* = 11–16/treatment) but with less of an impact on neuronal excitability (*post hoc* Sidak’s multiple-comparisons test, treatment effect: 200 pA, *p* = 0.4772; 300 pA, *p* = 0.2584; 400 pA, *p* = 0.0399; 500 pA, *p* = 0.0139). The resting membrane potential of DRG neurons in the *SNS-Nedd4L*^−/−^ mice decreased at more negative values after the treatment with metformin (control vs metformin: *p* = 0.0341, *t*_(19.18)_ = 2.282, *n* = 10–16/group), whereas the rheobase significantly increased (control vs metformin: *p* = 0.02, *t*_(21.56)_ = 2.513, *n* = 10–16/group), suggesting that metformin affects DRG excitability independent of NEDD4-2 (Fig. 5*B*). On the other hand, in the *Nedd4L^fl/fl^* mice the treatment of cultured DRG neurons with metformin significantly reduced the amplitude (control vs metformin: *p* = 0.0032, *t*_(24.04)_ = 3.278, *n* = 14/group), maximum rise time (control vs metformin: *p* = 0.0131, *t*_(22.5)_ = 2.693, *n* = 14/group), and baseline of individual APs (control vs metformin: *p* = 0.0043, *t*_(25.93)_ = 3.127, *n* = 14/group; Fig. 5*B*). The AP parameters, analyzed from recordings of *Nedd4L^fl/fl^* or *SNS-Nedd4L*^−/−^ DRG neurons with or without metformin treatment, are shown in Extended Data Figure 5-1. Together, our results suggest that mechanisms reducing the excitability of DRG neurons are partially dependent on NEDD4-2, but also independent of the ubiquitin ligase.

10.1523/ENEURO.0409-21.2022.f5-1 Figure 5-1 Supplemental data table supporting Figure 5. AP characteristics recorded from DRG neurons by electrophysiological current-clamp protocol. Neurons were obtained from *Nedd4L^fl/fl^* mice (*n* = 14 cells/treatment) or *SNS-Nedd4L^–/–^* mice (*n* = 10–16 cells/treatment) and treated with control medium or metformin treatment (20 mm). Data are represented as the mean ± SEM. Statistical analysis was performed using and unpaired *t* test with Welch’s correction (Extended Data Table 1-2). *p < 0.05, **p < 0.01, ***p < 0.001. Detailed references for the resources used are presented in Extended Data Table 1-1. Download Figure 5-1, XLSX file.

## Discussion

Despite the recent development of Na_V_1.7-specific inhibitors, small molecules, anti-Na_V_1.7 antibodies, or other new Na_V_ isoform inhibitors (Alsaloum et al., 2020), alternative strategies could be represented by post-translational regulation of the channel activity for the treatment of chronic pain (Laedermann et al., 2015). Metformin has this potential and offers the advantage of being used in clinical settings (Asiedu et al., 2016; Demaré et al., 2021). We demonstrate here that metformin induces a decrease in Na_V_1.7 channel expression through NEDD4-2 and ultimately reduces the excitability of the nociceptive neuron.

Metformin can enhance the NEDD4-2-mediated epithelial Na^+^ channel ENaC internalization, in an AMPK signaling-dependent manner (Bhalla et al., 2006). AMPK induces ENaC inhibition through the stabilization of the protein complex including NEDD4-2, thus producing ENaC retrieval from the plasma membrane, in which the binding motif in the cytoplasmic tail of the β-ENaC subunit is essential for the interaction with the ubiquitin ligase NEDD4-2 (Bhalla et al., 2006; Asher et al., 2003). Similarly, we found increased AMPK phosphorylation with metformin in HEK293 cells, as well as in cultured DRG neurons, and we observed that NEDD4-2 is essential for the metformin effect on Na_V_1.7 expression at the plasma membrane and Na_V_1.7 current density. Ultimately, restoring NEDD4-2 expression—for example, in experimental neuropathic pain—with a recombinant adeno-associated viral vector reduced the *I*_Na_ current densities in DRG sensory neurons and repressed the development of mechanical allodynia following SNI (Laedermann et al., 2013).

Although the mechanisms linking metformin to AMPK phosphorylation have been well investigated, this drug is a promiscuous compound that affects multiple pathways. For its efficacy, metformin requires the protein-threonine kinase LKB1, which phosphorylates and activates AMPK (Woods et al., 2003). In addition, another upstream kinase of AMPK is Ca^2+^/calmodulin-dependent protein kinase (CaMKK), which, when inhibited pharmacologically or downregulated by using RNA interference, abolished AMPK activation (Woods et al., 2005). Thus, metformin may act on different intracellular pathways, independent of AMPK, inducing changes in transcription factors and ion channel activity. For example, the pleiotropic effect of metformin was investigated in relation to the phosphorylation of different amino acids, but also with its interaction with the putative kinase targets such as SGK1 (serine/threonine-protein kinase), MAP2K2 (dual-specificity mitogen-activated kinase kinase 2), MAPK14 (mitogen activated protein kinase 14), CDK7 (cyclin-dependent kinase 7), and EGFR (epidermal growth factor receptor), raising questions related to their downstream chain of events (Hart et al., 2016). In our study, we observed a decreased level of NEDD4-2 phosphorylated at Ser-328. This site has been found to be phosphorylated by SGK1 (Debonneville et al., 2001; Flores et al., 2005). Flores et al. (2005) found that the phosphorylation of NEDD4-2 via SGK1 led to an impairment of ENaC–NEDD4-2 interaction and consequently to more channels at the cell surface. Interestingly, Bhalla et al. (2006) showed an AMPK-dependent modulation of NEDD4-2 controlling ENaC activity; however, they found that the SGK1 pathway was not involved in the AMPK regulation of ENaC. Because the levels of phosphorylated Ser-328 were altered, our results suggest that the SGK-1 pathway could be involved in this effect. It would be an interesting pathway to explore in parallel to AMPK signaling, especially because the sites of NEDD4-2 phosphorylated by AMPK are not well identified (Arévalo, 2015).

In cultured sensory trigeminal (TG) neurons, metformin induces an increase in AMPK activation and the inhibition of the mTOR pathway (Melemedjian et al., 2011). Metformin, as well as AMPK activators, can abrogate the phosphorylation of mTOR, 4EBP, and rS6 (for which phosphorylation is increased after nerve injury). The authors also suggested that pharmacological AMPK activation (including by using metformin) negatively normalizes aberrant translational control after peripheral nerve injury, a phenomenon that contributes to reduced pain hypersensitivity in experimental models of neuropathic pain (SNI and SNL).

Metformin could act transcriptionally and post-translationally to decrease neuronal excitability. Interestingly, AMPK activators could potentially have similar mechanisms, as for the allosteric modulator A769662: acute application of this compound blocked in a dose-dependent manner voltage-gated sodium channels in rat TG neurons primary cultures (Asiedu et al., 2017). The effect of metformin via NEDD4-2 could also occur through the modification of protein expression. We found that the amount of Na_V_1.7 in whole-cell lysates of cultured DRG neurons was decreased after metformin treatment, and this regulation was dependent on NEDD4-2. Thus, NEDD4-2 acts on other regulatory machinery of membrane protein internalization, and this could happen directly through an enzymatic post-translational ubiquitination or indirectly. Interestingly, Grimsey et al. (2015) described an atypical role for NEDD4-2 E3 ubiquitin ligase. They showed that different G-protein-coupled receptors could stimulate p38–MAPK activation via NEDD4-2-mediated ubiquitination (Grimsey et al., 2015). Thus, NEDD4-2 may also regulate ion channel protein expression via p38–MAPK signaling. Consequently, it was shown that metformin regulates the remodeling of the small conductance calcium-activated potassium channels (SK2 and SK3) in the atrial tissues of a rat model of type 2 diabetes mellitus through the inhibition of NOX4 expression (constitutive NAPDH oxidase), but also by significantly suppressing the p38–MAPK signaling pathway (Liu et al., 2018).

Regulation of *I*_Na_ was also investigated in relation with metformin treatment and interactions with cytoskeleton binding proteins. In the human breast cancer MCF-7 cell line, metformin decreases phosphorylation of cofilin, an actin-binding protein involved in cytoskeleton dynamics, which is upstream regulated by LIM-kinase and Rho-kinase, suggesting a possible interaction of metformin with Rho-kinase (Özdemİr and Ark, 2021). Furthermore, in chick DRG neurons Rho-kinase phosphorylates the collapsin response mediator protein 2 (CRMP2), and inactivates the ability of CRMP2 to promote microtubule assembly and Numb-mediated endocytosis during growth cone collapse (Arimura et al., 2005). Beside the complex with Numb, CRMP2 interacts also with Eps15 and NEDD4-2 to promote the endocytosis of Na_V_1.7 as a specific silencing of these targets changed the sodium currents (Dustrude et al., 2016; Cai et al., 2021; Gomez et al., 2021). But this appears only in relation with the CRMP2 post-translational modification state of SUMOylation. Therefore, we do not exclude this pathway by which metformin, mediated by Rho-kinase and CRMP2, may interact with NEDD4-2 to promote Na_V_1.7 internalization and loss of sodium currents.

AMPK acts on NEDD4-2 and consequently on the ion channels expressing the intracellular binding motif. This fact confirms our data on sodium current densities in both the heterologous and homologous primary systems in which treatment with metformin decreased the total *I*_Na_ and Na_V_1.7 currents with only a marginal effect on the biophysical properties of the channel. The modulation by metformin determined by NEDD4-2, however, is not only restricted at sodium channels. Treatment of mouse polarized kidney cortical cells with metformin inhibits K_V_7.1 activity by promoting NEDD4-2-dependent channel ubiquitination (Alzamora et al., 2010). Interestingly, the expression and current levels of K_V_1.4 are downregulated in an AMPK–PKC manner independent of NEDD4-2 or the related ubiquitin ligase NEDD4-1, and independent of the phosphoinositide 3-kinase (PI3K)–SGK1 signaling pathway, suggesting that NEDD4-2 probably exerts different functions depending on the binding motif of the cytoplasmic tail of ion channels (Andersen et al., 2018).

Our current-clamp data are in line with previously published studies indicating that metformin can reduce neuronal excitability, lowering the frequency of APs (Melemedjian et al., 2011). Na_V_1.7 is one of the most important members of the fast kinetics tetrodotoxin-sensitive sodium channels that contributes to the rising phase of APs (Bennett et al., 2019). Therefore, metformin treatment, lowering Na_V_1.7 expression, reduces the amplitude and maximum rise time of single APs, whereas the baseline is higher. We have shown that metformin is also able to partially reduce neuronal excitability in NEDD4-2 knock-out animals, alongside changes in the rheobase and RMP, indicating a possible effect through different pathways and channels.

Finally, a recent study showed that the effect of metformin was not limited to peripheral neurons, but metformin treatment also altered the activation of spinal microglia and astrocytes in male mice after SNI (Inyang et al., 2019). The authors described several mechanisms of action of metformin on the androgen-dependent regulation of OCT2 (organic cation transporter-2), but the possibility that metformin could also regulate microglial ion channels should not be excluded. Indeed, different potassium currents are increased in activated microglia (e.g., Kir2.1-mediated currents; Madry et al., 2018; Gattlen et al., 2020), which are also regulated by NEDD4-2, and could be the target of metformin.

Our study was not conceived to answer the question of a gender effect during metformin treatment, but the issue is of particular interest. The link between the cytoskeleton binding protein CRMP2 SUMOylation and Na_V_1.7 showed a reduction of expression and currents in female mice compared with males (Moutal et al., 2020). Moreover, knocking down the expression of NEDD4-2 with silencing RNA in DRG sensory neurons restored the loss of sodium currents, in CRMP2 SUMO-null knock-in (CRMP2K374A/K374A) female mice (Gomez et al., 2021). Apart from the link among CRMP2, NEDD4-2, and Na_V_1.7 that is gender different, the treatment with metformin reverses SNI-induced mechanical and cold hypersensitivity in male but not in female mice. This effect might be due, at least in part, to how metformin differently activated microglial cells between the two genders, whereas AMPK activity increases in DRG neurons in both male and female (Inyang et al., 2019). Whether changes into AMPK activity, NEDD4-2, and Na_V_1.7 currents after metformin administration is gender dependent need to be further investigated.

In conclusion, we have provided novel data regarding the mechanism of action of metformin on sodium channels, which play a crucial role in physiological and pathologic pain. Comprehension of those mechanisms opens new alternatives for the diminution of hyperexcitability related to neuropathic pain, in particular by targeting post-translational regulation of sodium channels.

## Synthesis

Reviewing Editor: Lindsay De Biase, University of California Los Angeles Decisions are customarily a result of the Reviewing Editor and the peer reviewers coming together and discussing their recommendations until a consensus is reached. When revisions are invited, a fact-based synthesis statement explaining their decision and outlining what is needed to prepare a revision will be listed below. The following reviewer(s) agreed to reveal their identity: NONE.

In this manuscript the authors use functional assays and western blot analysis to show that metformin can indirectly modulate the functional activity of NaV1.7 sodium channels. They reveal that metformin effects on NaV1.7 can reduce membrane excitability in sensory neurons through an AMPK-Nedd4-2 dependent pathway, although they also present evidence that some effects of metformin on NaV1.7 are Nedd4-2 independent. Elucidating these mechanisms underlying metformin effects on membrane excitability of sensory neurons is significant because it may facilitate development of more potent drugs to treat chronic and neuropathic pain. Both reviewers agree that the manuscript presents solid experimental results and is well written and easy to follow.

However, minor concerns were raised that need to be addressed.

1. In the abstract there is a typo in line 9 where “of” should be removed from “membrane fraction of is only reduced.”

2. Also in the abstract “biotinylated HEK cells” doesn’t seem like appropriate phrasing. Shouldn’t this be “Using biotinylation experiments in HEK cells” or “biotinylation approaches in HEK cells”?

3. Also in the abstract, given that metformin’s impact on NaV1.7 channels is not exclusively mediated by Nedd4-2, the word “essential” in line 18 should be changed to “critical” or “central.”

4. For the electrophysiological traces in Figure 1A and Figure 2A, the authors should consider not showing current responses to every other voltage step. There are so many overlapping current traces that it is somewhat difficult to appreciate what individual traces look like. It would also be helpful to add a small cartoon/diagram indicating the voltages steps that were used to generate these current responses.

5. In Figure 1B quantification, it appears that current densities are generally larger for the Nedd4-2 with mutated catalytic site compared to the no-Nedd4-2 condition. Why is this?

6. In figure 2B, were the ProTxII sensitive currents subtracted from the total sodium currents shown in figure 1A? Please indicate.

7. In the representative western blots shown in Figure 3, the effects of metformin are not at all obvious by eye (e.g. tubulin levels look comparable across the cells and any difference in NaV1.7 is barely perceptible if at all). Do they have other representative blots where the effects of metformin are at least a tiny bit perceptible to the eye? Why is there so much more variability in the detected levels of NaV1.7 in the -/- mice (regardless of whether metformin is applied)?

8. In figure 3, it appears that in DRG there is only core-glycosylated Nav1.7 channels and no fully glycosylated form of Nav1.7. Can the authors explain this difference in glycosylation between HEK cells and DRGs in more detail?

9. It has been reported that when CRMP2 is not SUMOylated it interacts with the endocytic machinery complex composed of Numb, Nedd4-2 and Eps15 to induce the endocytosis of NaV1.7 channels and sodium currents are reduced. However, when Nedd4-2 is absent in DRG neurons the loss of sodium currents is restored. Also, metformin inhibits the activation of Rho in MCF-7 cells, and it has been shown that inhibition of Rho kinases affects the function of CRMP2. Can this be contributing to the reduction of NaV1.7 currents and excitability after metformin treatment? Please include a brief discussion.

10. The Inyang paper cited in the discussion shows a strong sex difference in the effect of metformin in mice in a neuropathic pain model. The authors used male and female mice. Did the authors see any sex differences in their experiments? Please describe in the discussion.

## Author Response

Author’s Rebuttal

1. In the abstract there is a typo in line 9 where “of” should be removed from “membrane fraction of is only reduced.”

We thank the reviewer for pointing out this typo, which has been corrected in the final manuscript.

2. Also in the abstract “biotinylated HEK cells” doesn’t seem like appropriate phrasing. Shouldn’t this be “Using biotinylation experiments in HEK cells” or “biotinylation approaches in HEK cells”?

The sentence has been revised (using biotinylation approach) in the final document.

3. Also in the abstract, given that metformin’s impact on NaV1.7 channels is not exclusively mediated by Nedd4-2, the word “essential” in line 18 should be changed to “critical” or “central.”

The word “essential” has been replaced by “critical” in the manuscript.

4. For the electrophysiological traces in Figure 1A and Figure 2A, the authors should consider not showing current responses to every other voltage step. There are so many overlapping current traces that it is somewhat difficult to appreciate what individual traces look like. It would also be helpful to add a small cartoon/diagram indicating the voltages steps that were used to generate these current responses.

We agree with the comment. To improve Figures 1A and 2A, only one trace, corresponding to the maximum peak current is represented in Figures 1 and 2. In order to provide all the information, we show the representative electrophysiological traces for each voltage step in separate figures (extended Data Figure 1-2 and extended Data Figure 2-1), including a schematic representation of the step protocol applied.

5. In Figure 1B quantification, it appears that current densities are generally larger for the Nedd4-2 with mutated catalytic site compared to the no-Nedd4-2 condition. Why is this?

We agree with the reviewer. It is suggested in the literature that a competitive binding effect on the PY motif occurs between endogenous NEDD4-2 and NEDD4-2 CS (Rougier et al. 2005; Abriel et al. 2000). Rougier et al. (2005) and Abriel et al. (2000) have shown that an inactive NEDD4-2 CS mutant increases Nav1.5 current in cell expression system (HEK293 cells and Xenopus respectively). Similarly, Laedermann et al (2013) observed an increase of Nav1.7 current when co-transfected with NEDD4-2 CS (Laedermann et al. 2013a). This hypothesis was further supported by other data: when shNedd4-2 is co-transfected with Nav1.7 in HEK293 cells, it also leads to an increase of Nav1.7-mediated current (Supplemental Figure 2 Laedermann et al).

Our experimental design was not constructed to answer this question and we have chosen not to comment on it in the text.

6. In figure 2B, were the ProTxII sensitive currents subtracted from the total sodium currents shown in figure 1A? Please indicate.

The Protoxin II was applied solely for the recordings in the DRG neurons. We have now added this in the method section.

7. In the representative western blots shown in Figure 3, the effects of metformin are not at all obvious by eye (e.g. tubulin levels look comparable across the cells and any difference in NaV1.7 is barely perceptible if at all). Do they have other representative blots where the effects of metformin are at least a tiny bit perceptible to the eye?

According to the request of the reviewer, we have now modified Figure 3 with a blot that is more representative.

Why is there so much more variability in the detected levels of NaV1.7 in the -/- mice (regardless of whether metformin is applied)?

We hypothesize that a part of the variability might be due to NEDD4-2 protein expression in the knockout animals, which further modulate Nav1.7 expression. NEDD4-2 is knocked out only in cell expressing Nav1.8 (Cre recombinase under Nav1.8 promotor) and the expression of NEDD4-2 might be variable in between animals.

Previous reports suggested that the Cre recombinase efficiency might exhibit in some cases inter-individual variability within a transgenic mouse line (Heffner et al. 2012; Schmidt-Supprian and Rajewsky 2007). NEDD4-2-floxed mouse lines, including the one used in the current study, have been extensively used to decipher NEDD4-2 physiological function (Shi et al. 2008; Manning and Kumar 2018). Similarly, the SNS-Cre line, used in our study, has been used to target Nav1.8 positive neurons within the DRG (Agarwal et al. 2004). To our knowledge, such variability was never studied in detail, but it can be suggested based on our results.

8. In figure 3, it appears that in DRG there is only core-glycosylated Nav1.7 channels and no fully glycosylated form of Nav1.7. Can the authors explain this difference in glycosylation between HEK cells and DRGs in more detail?

The glycosylation of Nav1.7 channels is highly dependent on the presence of multiple intracellular partners. The co-expression of β subunits with the Navs has been previously described to play an essential role in the glycosylated state of the channel (Sokolov et al. 2018). In our study, the two bands observed in the HEK cells in the biotinylated fraction (Figure 1C) corresponds to two different glycosylated forms of Nav1.7. This is consistent with what have been previously shown in HEK cells in absence of β subunits (Laedermann et al. 2013b). In that study, the co-expression of β subunits with Nav1.7 in HEK cells, particularly β1 or β3, has been shown to shift the functional Nav1.7 form (fully glycosylated, upper band) toward an intermediate migrating suggested that Nav1.7 establish a stable complex with β1 and β3 subunits within the peripheral sensory neurons (Shah et al. 2000; Ho et al. 2012). In our study we observed a single band of Nav1.7 (Figure 3A and 3C) in DRG neurons which suggest that the difference in Nav1.7 glycosylation states between HEK cells and DRG neurons is due to differential expression of intracellular partners, especially the β subunits (not co-expressed in HEK cells experiments).

9. It has been reported that when CRMP2 is not SUMOylated it interacts with the endocytic machinery complex composed of Numb, Nedd4-2 and Eps15 to induce the endocytosis of NaV1.7 channels and sodium currents are reduced. However, when Nedd4-2 is absent in DRG neurons the loss of sodium currents is restored. Also, metformin inhibits the activation of Rho in MCF-7 cells, and it has been shown that inhibition of Rho kinases affects the function of CRMP2. Can this be contributing to the reduction of NaV1.7 currents and excitability after metformin treatment? Please include a brief discussion.

In the experiments of Özdemİr and Ark 2021, the authors claimed that 1h incubation with metformin decreased significantly the phosphorylation of cofilin (actin-binding protein involved in cytoskeleton dynamics) in MCF-7 cells (human breast cancer cells) suggesting that metformin suppresses the activation of Rho-kinase (ROCK) (Özdemi̇r and Ark 2021). The pathway they proposed is that once ROCK is activated, it phosphorylates LIM-kinase. Then LIM-kinase phosphorylates cofilin at serine 3 and inactivates the protein. In the study of Arimura et al, 2005 in chick DRG neurons, the authors showed that Rho-kinase phosphorylates CRMP2, which in turn inactivates the ability of CRMP2 to promote microtubule assembly and Numb-mediated endocytosis during growth cone collapse (Arimura et al. 2005). To our knowledge, it remains to be further explored how CRMP2 phosphorylation by Rho-kinase affects the interactions of CRMP2 with other partners, especially NEDD4-2. Additionally, it would be interesting to further investigate whether it directly contributes to the endocytosis of Nav1.7 in DRG neurons.

As mentioned by the reviewer, Gomez et al, 2021 demonstrated that regulation of Nav1.7 function follows CRMP2′s post-translational modification states, primarily phosphorylation and SUMOylation (i.e. addition of a small ubiquitin like modifier (SUMO)) (Gomez et al. 2021). When not SUMOylated, CRMP2 forms a complex with Numb, Eps15, and NEDD4-2 to promote the endocytosis of Nav1.7 (Dustrude et al. 2016). Silencing NEDD4-2 in DRG neurons from female CRMP2K374A/K374A mutant mice (SUMO-null knock-in), restored the loss of sodium currents. Finally, as shown recently, inhibition of SUMOylation of CRPM2 decreases NaV1.7 and pain hypersensitivity in experimental models of acute and chronic pain (Cai et al. 2021).

In our current study, we observed that metformin induced a change in the phosphorylation of NEDD4-2 on the Serine328 residue. This suggests that metformin acts on NEDD4-2 activity through the regulation of kinase activity. Nevertheless, further investigations exploring the effect of metformin on CRMP2 SUMOylation/Phosphorylation state and on its interactions with other molecular partners, notably with NEDD4-2, would be particularly interesting and relevant.

The action of metformin on Rho-kinase was shown to be changed in a human breast cancer cell line, then Rho-kinase phosphorylates CRMP2 in chicken neurons and CRMP2 interacts with NEDD4-2 to promote Nav1.7 internalization in a transgenic mouse line. Despite these evidence in various systems, we cannot exclude the metformin - Rho-kinase - CRMP2 - NEDD4-2 - Nav1.7 pathway until further work will be done. This is the reason we added in the discussion the following paragraph (Line 433-446):

Regulation of INa was also investigated in relation with metformin treatment and interactions with cytoskeleton binding proteins. In human breast cancer MCF-7 cell line metformin decreases phosphorylation of cofilin, an actin-binding protein involved in cytoskeleton dynamics, which is up-stream regulated by LIM-kinase and Rho-kinase, suggesting a possible interaction of metformin with Rho-kinase (Özdemi̇r and Ark 2021). Furthermore, in chick DRG neurons Rho-kinase phosphorylates the collapsin response mediator protein 2 (CRMP2), and inactivates the ability of CRMP2 to promote microtubule assembly and Numb-mediated endocytosis during growth cone collapse (Arimura et al. 2005). Beside the complex with Numb, CRMP2 interacts also with Eps15 and NEDD4-2 to promote the endocytosis of Nav1.7 as a specific silencing of these targets changed the sodium currents (Gomez et al. 2021; Dustrude et al. 2016; Cai et al. 2021). But this appears only in relation with the CRMP2′s post-translational modification state of SUMOylation. Therefore, we do not exclude this pathway by which metformin, mediated by Rho-kinase and CRMP2, may interact with NEDD4-2 to promote Nav1.7 internalization and loss of sodium currents.

10. The Inyang paper cited in the discussion shows a strong sex difference in the effect of metformin in mice in a neuropathic pain model. The authors used male and female mice. Did the authors see any sex differences in their experiments? Please describe in the discussion.

We thank the reviewer for raising this important question. When experiments were designed to understand metformin effect on Nav1.7 channels through NEDD4-2, we have not considered the gender as a potential confounding variable since to the best of our knowledge, NEDD4-2 is not known to be differently expressed in male vs female. In the Inyang paper, AMPK was equally activated in male and female, and the sex-specific effect of metformin was described to be due to microglia activation specifically in male. Other papers described differential microglial activation between male and female in neuropathic pain in mice and rats as well (Sorge et al. 2015; Taves et al. 2016). Surely, this would be of interest in further studies since some paper highlighted that Navs, including Nav1.7, might be differently regulated based on the sex (Moutal et al. 2020) and whether NEDD4-2 is involved in this differential regulation remains to be investigated.

For the current study, we used both male and female mice, but since the ratio for the different conditions is not well balanced (male or female were randomly picked), some sub-groups would have too few recordings or samples to make any assumption on a potential differential effect between males and females. Our experiments were not designed to answer this question, and a sub analysis of our data do not allow to make strong hypothesis on whether there is a sex difference in the proposed mechanisms. The question remains of large interest and we have added the following paragraph in the discussion.

Our study was not conceived to answer the question of a gender effect upon metformin treatment, but the issue is of particular interest. The link between the cytoskeleton binding protein CRMP2 SUMOylation and Nav1.7 showed a reduction of expression and currents, in female mice compared with males (Moutal et al. 2020). Moreover, knocking-down the expression of NEDD4-2 with silencing RNA in DRG sensory neurons restored the loss of sodium currents, in CRMP2 SUMO-null knock-in (CRMP2K374A/K374A) female mice (Gomez et al. 2021). Apart the link between CRMP2, NEDD4-2 and Nav1.7 that is gender different, the treatment with metformin reverses SNI-induced mechanical and cold hypersensitivity in male but not in female mice. This effect might be due, at least in part, to how metformin differently activated microglial cells between the two genders whereas AMPK activity increases in DRG neurons both in male and female (Inyang et al. 2019). Whether changes into AMPK activity, NEDD4-2 and Nav1.7 currents after metformin is gender dependent need to be further investigated.

References

Abriel, H., E. Kamynina, J. D. Horisberger, and O. Staub. 2000. ’Regulation of the cardiac voltage-gated Na+ channel (H1) by the ubiquitin-protein ligase Nedd4’, FEBS Lett, 466: 377-80.

Agarwal, N., S. Offermanns, and R. Kuner. 2004. ’Conditional gene deletion in primary nociceptive neurons of trigeminal ganglia and dorsal root ganglia’, Genesis, 38: 122-9.

Arimura, Nariko, CéLine MéNager, Yoji Kawano, Takeshi Yoshimura, Saeko Kawabata, Atsushi Hattori, Yuko Fukata, Mutsuki Amano, Yoshio Goshima, Masaki Inagaki, Nobuhiro Morone, Jiro Usukura, and Kozo Kaibuchi. 2005. ’Phosphorylation by Rho Kinase Regulates CRMP-2 Activity in Growth Cones’, Molecular and Cellular Biology, 25: 9973-84.

Cai, S., A. Moutal, J. Yu, L. A. Chew, J. Isensee, R. Chawla, K. Gomez, S. Luo, Y. Zhou, A. Chefdeville, C. Madura, S. Perez-Miller, S. S. Bellampalli, A. Dorame, D. D. Scott, L. Francois-Moutal, Z. Shan, T. Woodward, V. Gokhale, A. G. Hohmann, T. W. Vanderah, M. Patek, M. Khanna, T. Hucho, and R. Khanna. 2021. ’Selective targeting of NaV1.7 via inhibition of the CRMP2-Ubc9 interaction reduces pain in rodents’, Sci Transl Med, 13: eabh1314.

Dustrude, Erik T., Aubin Moutal, Xiaofang Yang, Yuying Wang, May Khanna, and Rajesh Khanna. 2016. ’Hierarchical CRMP2 posttranslational modifications control NaV1.7 function’, Proceedings of the National Academy of Sciences, 113: E8443-E52.

Gomez, Kimberly, Dongzhi Ran, Cynthia L. Madura, Aubin Moutal, and Rajesh Khanna. 2021. ’Non-SUMOylated CRMP2 decreases NaV1.7 currents via the endocytic proteins Numb, Nedd4-2 and Eps15’, Molecular Brain, 14.

Heffner, Caleb S., C. Herbert Pratt, Randal P. Babiuk, Yashoda Sharma, Stephen F. Rockwood, Leah R. Donahue, Janan T. Eppig, and Stephen A. Murray. 2012. ’Supporting conditional mouse mutagenesis with a comprehensive cre characterization resource’, Nat Commun, 3: 1218.

Ho, Cojen, Juan Zhao, Steven Malinowski, Mohamed Chahine, and Michael E. O’Leary. 2012. ’Differential Expression of Sodium Channel β Subunits in Dorsal Root Ganglion Sensory Neurons’, Journal of Biological Chemistry, 287: 15044-53.

Inyang, K. E., T. Szabo-Pardi, E. Wentworth, T. A. McDougal, G. Dussor, M. D. Burton, and T. J. Price. 2019. ’The antidiabetic drug metformin prevents and reverses neuropathic pain and spinal cord microglial activation in male but not female mice’, Pharmacol Res, 139: 1-16.

Laedermann, C. J., M. Cachemaille, G. Kirschmann, M. Pertin, R. D. Gosselin, I. Chang, M. Albesa, C. Towne, B. L. Schneider, S. Kellenberger, H. Abriel, and I. Decosterd. 2013a. ’Dysregulation of voltage-gated sodium channels by ubiquitin ligase NEDD4-2 in neuropathic pain’, J Clin Invest, 123: 3002-13.

Laedermann, C. J., N. Syam, M. Pertin, I. Decosterd, and H. Abriel. 2013b. ’beta1- and beta3- voltage-gated sodium channel subunits modulate cell surface expression and glycosylation of Nav1.7 in HEK293 cells’, Front Cell Neurosci, 7: 137.

Manning, J. A., and S. Kumar. 2018. ’Physiological Functions of Nedd4-2: Lessons from Knockout Mouse Models’, Trends Biochem Sci, 43: 635-47.

Moutal, Aubin, Song Cai, Jie Yu, Harrison J. Stratton, Aude Chefdeville, Kimberly Gomez, Dongzhi Ran, Cynthia L. Madura, Lisa Boinon, Maira Soto, Yuan Zhou, Zhiming Shan, Lindsey A. Chew, Kathleen E. Rodgers, and Rajesh Khanna. 2020. ’Studies on CRMP2 SUMOylation-deficient transgenic mice identify sex-specific Nav1.7 regulation in the pathogenesis of chronic neuropathic pain’, Pain, 161: 2629-51.

Özdemi̇r, Aysun, and Mustafa Ark. 2021. ’A novel ROCK inhibitor: off-target effects of metformin’, TURKISH JOURNAL OF BIOLOGY, 45: 35-45.

Rougier, J. S., M. X. van Bemmelen, M. C. Bruce, T. Jespersen, B. Gavillet, F. Apotheloz, S. Cordonier, O. Staub, D. Rotin, and H. Abriel. 2005. ’Molecular determinants of voltage-gated sodium channel regulation by the Nedd4/Nedd4-like proteins’, Am J Physiol Cell Physiol, 288: C692-701.

Schmidt-Supprian, Marc, and Klaus Rajewsky. 2007. ’Vagaries of conditional gene targeting’, Nature Immunology, 8: 665-68.

Shah, B. S., E. B. Stevens, M. I. Gonzalez, S. Bramwell, R. D. Pinnock, K. Lee, and A. K. Dixon. 2000. ’β3, a novel auxiliary subunit for the voltage-gated sodium channel, is expressed preferentially in sensory neurons and is upregulated in the chronic constriction injury model of neuropathic pain’, European Journal of Neuroscience, 12: 3985-90.

Shi, P. P., X. R. Cao, E. M. Sweezer, T. S. Kinney, N. R. Williams, R. F. Husted, R. Nair, R. M. Weiss, R. A. Williamson, C. D. Sigmund, P. M. Snyder, O. Staub, J. B. Stokes, and B. Yang. 2008. ’Salt-sensitive hypertension and cardiac hypertrophy in mice deficient in the ubiquitin ligase Nedd4-2’, Am J Physiol Renal Physiol, 295: F462-70.

Sokolov, Maxim V., Petra Henrich-Noack, Carina Raynoschek, Bo Franzén, Olof Larsson, Martin Main, and Michael Dabrowski. 2018. ’Co-expression of β Subunits with the Voltage-Gated Sodium Channel NaV1.7: the Importance of Subunit Association and Phosphorylation and Their Effects on Channel Pharmacology and Biophysics’, Journal of Molecular Neuroscience, 65: 154-66.

Sorge, R. E., J. C. Mapplebeck, S. Rosen, S. Beggs, S. Taves, J. K. Alexander, L. J. Martin, J. S. Austin, S. G. Sotocinal, D. Chen, M. Yang, X. Q. Shi, H. Huang, N. J. Pillon, P. J. Bilan, Y. Tu, A. Klip, R. R. Ji, J. Zhang, M. W. Salter, and J. S. Mogil. 2015. ’Different immune cells mediate mechanical pain hypersensitivity in male and female mice’, Nat Neurosci, 18: 1081-3.

Taves, S., T. Berta, D. L. Liu, S. Gan, G. Chen, Y. H. Kim, T. Van de Ven, S. Laufer, and R. R. Ji. 2016. ’Spinal inhibition of p38 MAP kinase reduces inflammatory and neuropathic pain in male but not female mice: Sex-dependent microglial signaling in the spinal cord’, Brain Behav Immun, 55: 70-81.
